# The loss of enzymatic activity of the PHARC‐associated lipase ABHD12 results in increased phagocytosis that causes neuroinflammation

**DOI:** 10.1111/ejn.15516

**Published:** 2021-11-11

**Authors:** Shubham Singh, Siddhesh S. Kamat

**Affiliations:** ^1^ Department of Biology Indian Institute of Science Education and Research (IISER), Pune Pune India

**Keywords:** ABHD12, cerebellum, lyso‐PS, microglia, neuroinflammation, neurons, oxidized PS, phagocytosis, PHARC

## Abstract

Phagocytosis is an important evolutionary conserved process, essential for clearing pathogens and cellular debris in higher organisms, including humans. This well‐orchestrated innate immunological response is intricately regulated by numerous cellular factors, important amongst which are the immunomodulatory lysophosphatidylserines (lyso‐PSs) and the pro‐apoptotic oxidized phosphatidylserines (PSs) signalling lipids. Interestingly, in mammals, both these signalling lipids are physiologically regulated by the lipase ABHD12, mutations of which cause the human neurological disorder PHARC. Despite the biomedical significance of this lipase, detailed mechanistic studies and the specific contribution of ABHD12 to innate processes like phagocytosis remain poorly understood. Here, by immunohistochemical and immunofluorescence approaches, using the murine model of PHARC, we show, that upon an inflammatory stimulus, activated microglial cells in the cerebellum of mice deficient in ABHD12 have an amoeboid morphology, increased soma size and display heightened phagocytosis activity. We also report that upon an inflammatory stimulus, cerebellar levels of ABHD12 increase to possibly metabolize the heightened oxidized PS levels, temper phagocytosis and, in turn, control neuroinflammation during oxidative stress. Next, to complement these findings, with the use of biochemical approaches in cultured microglial cells, we show that the pharmacological inhibition and/or genetic deletion of ABHD12 results in increased phagocytic uptake in a fluorescent bead uptake assay. Together, our studies provide compelling evidence that ABHD12 plays an important role in regulating phagocytosis in cerebellar microglial cells and provides a possible explanation, as to why human PHARC subjects display neuroinflammation and atrophy in the cerebellum.

## Graphical abstract

In mammals, the lipase ABHD12, mutations of which cause the human neurological disorder PHARC, metabolizes immunomodulatory lyso‐PS and pro‐apoptotic oxidized PS lipids. Here, with the use of the murine model of PHARC and cellular systems, we show, that upon an inflammatory stimulus, loss of ABHD12 activity results in heightened microglial phagocytosis activity. We also provide a possible explanation, as to why human PHARC subjects display neuroinflammation and cerebellar atrophy.

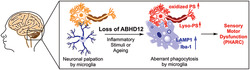

**ABBREVIATIONS**
ABHD12: α/β‐hydrolase domain containing protein # 12
ABHD16A: α/β‐hydrolase domain containing protein # 16A
LPS: lipopolysaccharide
Lyso‐PS: lysophosphatidylserine
Oxidized PS: oxidized phosphatidylserine
PHARC: Polyneuropathy, Hearing Loss, Ataxia, Retinitis Pigmentosa, Cataract
TLR2: toll‐like receptor 2

## 1 INTRODUCTION

Phagocytosis is an important innate immune response, conserved in both prokaryotes and eukaryotes (Flannagan et al., 2012). In early single cellular life forms, phagocytosis is an important way to obtain nutrients, whereas, in more evolved complex multicellular organisms, this physiologically indispensable response is central to an organism's immunity (Flannagan et al., 2012; Levin et al., 2016). In higher organisms, phagocytosis is the first type of defence against invading pathogens and is essential for clearing infections, or cellular debris (Flannagan et al., 2012; Murray & Wynn, 2011; Steinberg & Grinstein, 2008). In mammals (including humans), the phagocytic cells (typically macrophages or cells with a monocyte lineage) have an innate ability to recognize, engulf and digest foreign solid particles ≥ 500 nm (e.g., pathogens like viruses and bacteria) or any defective/toxic material generated during heightened metabolic states and/or injury (e.g., diseased cells, protein aggregates and tissue debris) with sufficient antigenicity (Murray & Wynn, 2011; Steinberg & Grinstein, 2008; Tauber, 2003). Often, a distressed (injured or infected or apoptotic) cell needs to be cleared from the circulatory system, to prevent any damage to its surrounding cells or tissues, and such a cell secretes substances (e.g., signalling lipids and chemokines) that provide phagocytic cells (e.g., circulating macrophages) signature signalling cues, to initiate its engulfment and clearance from the system via phagocytosis (Kinchen & Ravichandran, 2008; Levin et al., 2016; Martin et al., 2014; Murray & Wynn, 2011; Vieira et al., 2002). Amongst the signalling lipids thought to be involved in facilitating phagocytosis (Flannagan et al., 2012; Levin et al., 2016; Steinberg & Grinstein, 2008) are the immunomodulatory lysophosphatidylserines (lyso‐PSs) (Shanbhag et al., 2020) and the pro‐apoptotic oxidized phosphatidylserines (PSs) (Kelkar et al., 2019).

Of the many important immunological functions that the lyso‐PSs perform in mammalian physiology (Shanbhag et al., 2020), this signalling lipid in particular promotes efferocytosis (Doran et al., 2020; Elliott et al., 2017; Martin et al., 2014) and plays an important role in the resolution of inflammation by enhancing the phagocytic activity of macrophages (Frasch et al., 2008; Frasch & Bratton, 2012; Shanbhag et al., 2020). In mammalian macrophages, we have recently shown that the very long chain (VLC) lyso‐PSs activate these innate immune cells and elicit the secretion of pro‐inflammatory cytokines (e.g., tumour necrosis factor alpha [TNF‐α] and interleukin 6 [IL‐6]) via a toll‐like receptor 2 (TLR2) dependent pathway, whereas LC lyso‐PSs regulate various signalling cascades (cytosolic calcium influx, cyclic adenosine monophosphate production and phosphorylation of the nodal signalling protein extracellular signal‐regulated kinase [ERK]) possibly through an as‐of‐yet unknown G‐protein coupled receptor (GPCR) (Khandelwal et al., 2021). On the other hand, the pro‐apoptotic oxidized PSs have a long‐standing association with phagocytosis, particularly in the context of clearing up diseased/dying cells during oxidative stress or tissue injury (Greenberg et al., 2006; Kagan et al., 2002; Matsura et al., 2005; Tyurina et al., 2000). Given the asymmetric localization of almost all the PS to the inner leaflet of the membrane bilayer, the externalization of PS (due to its non‐enzymatic oxidation to yield oxidized PSs) represents a cell in oxidative stress and undergoing apoptosis (Greenberg et al., 2008; Hazen, 2008). This extracellularly facing PS from such distressed cells is an ‘eat me’ cue to circulating macrophages, whose scavenger receptors (e.g., CD36) recognize this externalized oxidized PS and initiate signalling pathways to start engulfing and digesting these apoptotic cells via phagocytosis (Greenberg et al., 2008; Hazen, 2008).

**FIGURE 1:**
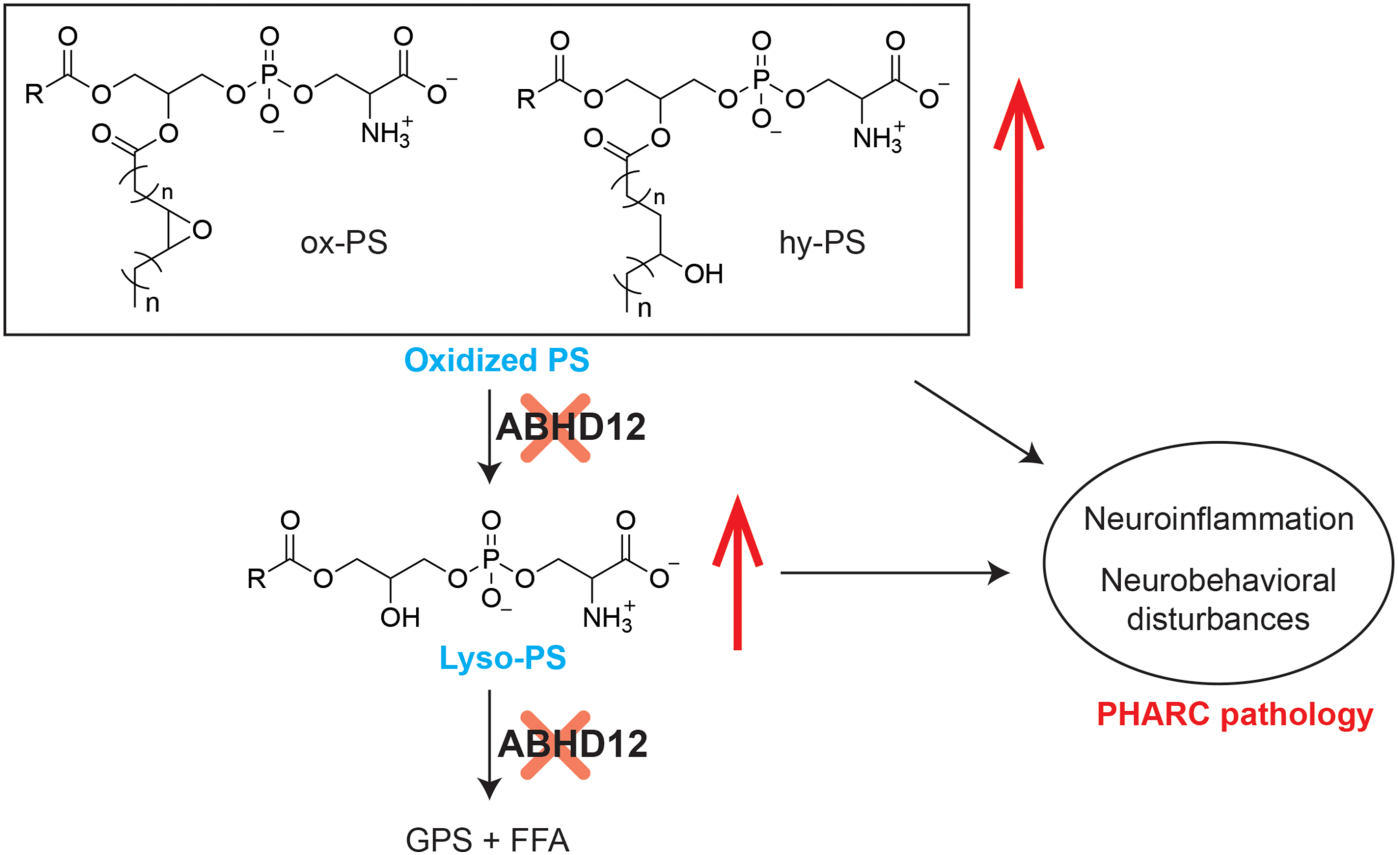
The biochemical pathways regulated by the lipase ABHD12. Loss of ABHD12 lipase activity causes accumulation of lysophosphatidylserine (lyso‐PS) (Blankman et al., 2013) and oxidized PS lipids (Kelkar et al., 2019) in the brain that contribute to neuroinflammation that manifests into the neurobehavioral defects associated with the human neurological disorder PHARC (Fiskerstrand et al., 2010). GPS = glycerophosphoserine and FFA = free fatty acid. This figure is adapted with modifications from a previous study from our lab (Kelkar et al., 2019)

Interestingly, in all mammals (including humans), the integral membrane lipase ABHD12 (α/β‐hydrolase domain containing protein # 12) from the metabolic serine hydrolase family (Joshi et al., 2018) controls the physiological concentrations of both lyso‐PSs (Blankman et al., 2013; Kamat et al., 2015) and oxidized PSs (Kelkar et al., 2019) in the central nervous system and various immune cells, including macrophages, particularly during conditions of oxidative stress (Figure 1). This metabolic activity of ABHD12 has significant biomedical importance, as deleterious (null) mutations to the gene encoding this lipase in humans results in an autosomal recessive early onset neurodegenerative disease called PHARC ( $\underset{―}{p}$olyneuropathy, ***h*** earing loss, ***a*** taxia, ***r*** etinitis pigmentosa and ***c*** ataract) (Figure 1) (Fiskerstrand et al., 2009; Fiskerstrand et al., 2010). Of note, immunohistochemical (IHC) and behavioural studies performed in ABHD12 knockout mice, the murine model of PHARC, show that the loss of enzymatic activity of ABHD12 is associated with an increase in the number of activated microglia (brain‐resident macrophages) and, in turn, neuroinflammation in the cerebellum (in the arbor vitae region) that eventually manifests in neurobehavioral defects (e.g., auditory and motor deficiencies) closely mimicking those observed in human PHARC subjects (Blankman et al., 2013). Additionally, lipidomics studies from cultured mammalian cells following the pharmacological and/or genetic disruption of ABHD12 have shown that the loss of ABHD12 activity causes the accumulation of both lyso‐PSs and oxidized PSs that results in increased activation of macrophages devoid of ABHD12 relative to wild type controls (Kamat et al., 2015; Kelkar et al., 2019).

Given that the enzymatic activity of ABHD12 controls physiological concentrations of the signalling lipids that are associated with the process of phagocytosis (Shanbhag et al., 2020), we wanted to assess if the loss of this lipase activity has any effect on phagocytosis and if this has any implications on our understanding of the pathology of the human neurological disorder PHARC (Fiskerstrand et al., 2010). Towards this, using IHC, immunofluorescence and biochemical approaches in ABHD12‐null mice and microglial cell lines, we show that the loss of ABHD12 activity results in heightened phagocytosis activity by (activated) microglial cells, and our studies taken together, perhaps provides a preliminary explanation, as to why aged ABHD12 knockout mice display neuroinflammation and human PHARC subjects show atrophy in their cerebellum.

## 2 MATERIALS AND METHODS

### 2.1 Materials

Unless mentioned otherwise, all chemicals, buffers and reagents described in this paper were purchased from Sigma‐Aldrich. All mammalian tissue culture media, consumables, antibiotics and foetal bovine serum (FBS) were purchased from HiMedia. All primary and secondary antibodies were purchased from Abcam and Thermo Fisher Scientific, respectively, unless stated otherwise. The fluorescent carboxylate modified microspheres (fluorescent latex beads) (Thermo Fisher Scientific, Catalog # F8813) were a kind gift from Prof. Roop Mallik (IIT Mumbai). The ABHD12 selective inhibitor JJH350 used in this study was a kind gift from Prof. Benjamin F. Cravatt (Scripps Research).

### 2.2 Animal studies

All animal (mouse) studies described in this paper have received formal approval from Institutional Animal Ethics Committee at IISER Pune (IAEC–IISER Pune) (application numbers: IISER_Pune IAEC/2019_02/07 and IISER_Pune IAEC/2021_01/09), constituted as per the guidelines outlined by the Committee for the Purpose of Control and Supervision of Experiments on Animals (CPCSEA), Government of India. All mice were bred and maintained at National Facility for Gene Function in Health and Disease (NFGFHD), IISER Pune. The ABHD12 knockout mice used in this study were generated on the C57BL/6‐genetic background as reported earlier (Blankman et al., 2013). All mice used in the study were generated by a standard ABHD12 heterozygous × ABHD12 heterozygous breeding scheme, genotyped using previously established protocols (Blankman et al., 2013), and had ad libitum access to food and water. For all the animal studies described here, an equal number of age‐matched male and female mice (10–12 weeks of age) were used to control for any gender and age specific effects. Prof. Benjamin F. Cravatt (Scripps Research) is thanked for gifting the ABHD12 heterozygous breeder mice used to generate the ABHD12 knockout mice used in this study. Previously, we have found that the high‐dose acute (10–20 mg/kg body weight, intraperitoneally, 4–6 h) intraperitoneal treatment with LPS produces the best genotypic differences in the extent of neuroinflammation between wild type and ABHD12 knockout mice (Kelkar et al., 2019; Khandelwal et al., 2021), and hence, LPS was dosed at 10 mg/kg body weight (intraperitoneally) for 4‐h throughout this study.

### 2.3 IHC and immunofluorescence sample preparations

All IHC experiments and analysis were done using protocols recently reported by us (Khandelwal et al., 2021; Singh et al., 2020). Briefly, wild type or ABHD12 knockout mice (10–12 weeks old, equal number of males and females used in any given experiment) were deeply anaesthetized using isoflurane and perfused, first with chilled phosphate‐buffered saline (PBS) and then with ice cold 4% (w/v) paraformaldehyde (PFA) in PBS. To ensure anatomical integrity, the brains were gently harvested and transferred to 4% (w/v) PFA in PBS overnight (∼16–18 h) at 4°C to ensure proper tissue fixation. Thereafter, the fixed brains were transferred into a 30% (w/v) sucrose solution in PBS and maintained at 4°C until the brains sank to the bottom of the tubes (∼3 days). Cryoprotected brains were sectioned in 25‐μm‐thick coronal sections on a Leica CM1950 cryotome maintained at −30°C. Every 5th cerebellar section was collected and stored in PBS at 4°C, followed by its processing for IHC experiments. For DAB‐based bright‐field IHC analysis, endogenous peroxidases were inactivated and/or quenched with 3% (v/v) hydrogen peroxide in PBS for 15 min at 25°C in the dark, following which the sections were washed twice with 1% (w/v) bovine serum albumin (BSA) in PBS and permeabilized with 0.1% (w/v) Triton X‐100 and 0.5% (w/v) BSA in PBS for 45 min at 25°C. Following this, the sections were incubated with the primary antibody (dilution 1:100) in 1% (w/v) BSA overnight (~12–16 h) at 4°C, washed three times with 0.5% (w/v) BSA in PBS and incubated with the secondary antibody, biotinylated horse anti‐rabbit (Vector laboratories, BP‐1100) or biotinylated goat anti‐mouse (Vector laboratories, BP‐9200) at a dilution of 1:1000, in 0.5% (w/v) BSA in PBS for 1 h at 25°C. Following this, the sections were washed thrice in 0.5% (w/v) BSA in PBS, incubated with ABC Elite Vectastatin (Vector laboratories, PK‐6100) for 1 h at 25°C and washed thrice with excess PBS at 25°C. Finally, the sections were stained with ImmPACT DAB (Vector laboratories, SK‐4105) in the dark for 3 min and washed five times with PBS to remove excess DAB. Thereafter, the slides were mounted using VectaMount (Vector laboratories, H‐5000). All the stained sections were imaged using under bright field channel using a Zeiss Apotome microscope. For fluorescence IHC analysis, comparable cerebellar sections were permeabilized with 0.1% (v/v) Triton X‐100 and 0.5% (w/v) BSA in PBS for 45 min at 25°C and blocked with 1% (w/v) BSA in PBS for 60 min at 25°C. Following this step, the sections were washed thrice with 0.5% (w/v) BSA in PBS and incubated overnight (~12–16 h) with the primary antibody (dilution 1:100) at 25°C. Thereafter, the sections were washed thrice with 0.5% (w/v) BSA in PBS and incubated with the secondary antibody (1:1000 dilution) for 60 min at 25°C. Sections were again washed with PBS (three times) and mounted with Fluoromount aqueous mounting medium (Sigma‐Aldrich, F4680). The stained sections were imaged using a Leica SP8 confocal laser scanning microscope. The primary antibodies used in these studies were anti‐ABHD12 (rabbit, monoclonal, Abcam, catalog ab182011), anti‐Iba1 (rabbit, monoclonal, Abcam, catalog ab178846), anti‐Iba1 (mouse, monoclonal, Santa Cruz Biotechnology, catalog sc‐32725) and anti‐LAMP1 (rabbit, polyclonal, Abcam, catalog ab24170). The following secondary antibodies used in this study were goat anti‐rabbit IgG (H&L) conjugated with DyLight 488 (Thermo Fisher Scientific, 35553) and goat anti‐mouse IgG (H&L) conjugated with Alexa Fluor 568 (Thermo Fisher Scientific, A‐11004).

### 2.4 Activated microglia counting and analysis in the IHC experiments

Using the Fiji‐ImageJ1.50i (National Institutes of Health [NIH], for Windows OS) software, all the 2‐D bright field images were converted to 8‐bit grey scale (Image<<Type<<8‐bit). The thresholding for these grey scale images was manually done to mark the soma of the microglia (Image<<Adjust<<Threshold), and the soma size (area) of the microglia was measured using particle analysis tool (Analyze>>Analyze Particles) with set output of area of the particles and their number. From the Iba‐1 immunostaining profiles, the microglia with soma size (area) > 200 μm^2^ were considered as activated as per previous reports (Blankman et al., 2013; Khandelwal et al., 2021), whereas those with soma size < 200 μm^2^ were considered non‐activated. For size comparisons between the two genotypes, soma size of all the activated microglia in both wild type and ABHD12 knockout mice from the different biological replicates were used in the analysis. Based on previous results for the neuroinflammation observed in ABHD12 null mice, the arbor vitae region near deep cerebellar nuclei in the cerebellum was used for this IHC analysis, because this region also has cleaner microglia staining with very minimal background of DAB, and the microglial morphology and number of microglial cells are also largely homogenous with less variation basally, compared with other anatomical regions in cerebellum (e.g., the granular layers or molecular layers of different cerebellar peduncles).

### 2.5 Quantification of fluorescent markers in the IHC experiments

For all the IHC experiments, 3‐D stacks were imaged and recorded on a confocal microscope, and all analysis was performed offline using Fiji‐ImageJ1.50i (NIH, for Windows OS) software. For quantitation, the image stack showing middle plane of the cells with visible nuclei in centre and least overlap of the microglia cytoplasmic marker (Iba1) with nucleus was chosen manually and saved as a coloured (RGB) image. Cell boundaries of microglia were marked manually using the Iba1 signal as reference, following which the channel of interest was extracted (green for LAMP1 or ABHD12), and the images of extracted channel were converted to 8‐bit scale. Thereafter, intensity of the protein of interest was quantified for the whole cell by manually selecting the cell boundary. In this experiment, at least 10 such cells were counted per mice (i.e., per biological replicates), and the average (normalized) intensity per mouse (i.e. biological replicate) was reported. As described previously, this analysis was performed in the arbor vitae region of the cerebellum, which also has the least fluorescent noise and the microglia of this anatomical region are morphologically homogenous.

### 2.6 Western blot analysis

All western blot analysis (immunoblotting) was done using protocols previously reported by us (Rajendran et al., 2020; Singh et al., 2020). Briefly, the protein lysates (50 μg) were resolved on a 12.5% SDS−PAGE gel and transferred onto a methanol‐activated PVDF membrane (GE Healthcare) at 60 V for 12 h at 4°C. Following the transfer, the membrane was incubated with the respective primary and secondary antibodies at 1:1000 and 1:10,000 dilutions, respectively, using standard protocols reported by us (Rajendran et al., 2020; Singh et al., 2020). The blots were developed using the Thermo West Pico Western blotting substrate (Thermo Fisher Scientific), and the images were recorded on a Syngene G‐Box Chemi‐XRQ gel documentation system. The following primary antibodies were used in this study: anti‐ABHD16A (rabbit, monoclonal, Abcam, 185549), anti‐ABHD12 (rabbit, monoclonal, Abcam, ab182011), anti‐Iba1 (mouse, monoclonal, Santa Cruz Biotechnology, catalog sc‐32725), anti‐GAPDH (rabbit, monoclonal, Abcam, catalog ab1818602) and anti‐β‐actin (rabbit, polyclonal, Cloud Clone Corp., CAB340Hu01). The following secondary antibodies were used in this study: HRP‐conjugated anti‐rabbit IgG (goat, Thermo Fisher Scientific, 31460) and HRP‐linked anti‐mouse IgG (goat, Cloud Clone Corp., SAA544Mu19). All densitometric analyses of bands detected in these immunoblotting experiments were performed using Fiji‐ImageJ 1.50i (NIH, for Windows OS) software (Rueden et al., 2017; Schindelin et al., 2015). To quantify the blots, lanes on the acquired western blot images were manually marked and selected, and intensity of the band was determined using ‘Plot Lanes’ option available under Analyse>>Gels in the software. Thereafter, the area under the curve (for intensity plot) was measured using the wand tracing tools. All intensities of proteins of interest were divided by intensity of marker control proteins per experiment, per replicate, and the ratios obtained from this were graphically plotted.

### 2.7 Mammalian cell lines culturing

The BV‐2 (RRID:CVCL_0182) and N9 (RRID:CVCL_0452) immortalized microglial cell lines were a kind gift from Prof. Anirban Basu (National Brain Research Centre, India). Both the cell lines were stained routinely with DAPI to ensure that they were devoid of any mycoplasma contamination. The N9 microglial cell line was cultured in RPMI1640 media supplemented with 10% (v/v) heat inactivated FBS and 1× antibiotics (penicillin–streptomycin, MP Biomedicals) at 37°C with 5% (v/v) CO_2_. The BV‐2 microglial cell line was cultured in DMEM media supplemented with 10% (v/v) heat inactivated FBS and 1× antibiotics (penicillin–streptomycin, MP Biomedicals) at 37°C with 5% (v/v) CO_2_. Primary microglia cells were isolated from brains of Day 18 (E18) embryos of mice as reported previously (Viader et al., 2016). Briefly, brains were dissected out of E18 embryos, and meninges were carefully stripped and removed. Thereafter, the brain was trypsinized using 0.25% (w/v) trypsin on ice for 10 min, followed by incubation with DNAse I (HiMedia ‐ ML068) for another 5 min on ice. Following this, the cells of the brain were pelleted at 300 g for 15 min at 4°C, suspended in DMEM media by pipetting and plated on poly‐D‐lysine coated glass coverslips. The cells were maintained for 3–5 days in DMEM media supplemented with 10% (v/v) heat inactivated FBS and 1× antibiotic‐antimycotic (Thermo Fisher Scientific, catalog # 15240096) at 37°C with 5% (v/v) CO_2_. The media for this microglial cell culture was changed every 2 days, and microglia cells were identified by anti‐Iba1 immunostaining. Primary thioglycollate elicited peritoneal macrophages were generated from mice and cultured using established protocols recently reported by us (Kelkar et al., 2019; Singh et al., 2020). For all the cellular experiments, the cells were counted using a Trypan Blue staining method on a BC20 automated cell counter (Bio‐Rad) as per manufacturer's instructions.

### 2.8 Phagocytosis assay

To measure the extent of phagocytosis by microglial cells and/or macrophages, established fluorescent latex bead engulfment assays were performed. For this bead engulfment assay, microglial cells (BV‐2 or N9) or primary microglia or macrophages were grown on poly‐D‐lysine coated glass coverslips to about 50–60% confluency. Thereafter, these cells were incubated with fluorescent carboxylate modified microspheres (fluorescent latex beads, 0.5 μm, Thermo Fisher Scientific, catalog # F8813) at a 1:200 cell to bead ratio, for 30 min at 37°C, 5% (v/v) CO_2_. Following the bead incubation step, the cells were washed with ice‐cold sterile Dulbecco's phosphate‐buffered saline (DPBS) and fixed with 4% (w/v) PFA in DBPS at ambient temperature (23–25°C) using established protocols (Rajendran et al., 2020; Singh et al., 2020) and subsequently imaged to count the number of beads engulfed by phagocytosis by the microglial cells and/or macrophages. The *N*‐hydroxyhydantoin‐carbamate JJH350, a ABHD12 specific covalent inhibitor (Ogasawara et al., 2019), was used for the pharmacological inhibition of ABHD12 in the bead engulfment assays, where the cells were treated with 10‐μM JJH350 for 6 h, following which the bead engulfment assay was performed as described above. All quantifications and image analysis were done offline using Fiji‐ImageJ1.50i (NIH, for Windows OS) software. For quantitation, image stack showing middle plane of the cell with visible nuclei in centre and least overlap of the cellular cytoplasmic marker (Iba1 and/or Phalloidin) with nucleus was chosen manually and saved as a coloured RGB image. Cell boundaries of microglia were marked manually using the Phalloidin signal as a reference, following which the channel of interest was extracted (green for beads), and the resulting images of extracted channel were converted to 8‐bit scale. Thereafter, the intensity threshold was manually adjusted using the software route (Image>>Color>>Threshold) to mark the beads, and these engulfed particles were analysed using analyze particles option (Analyze>>Analyze Particles) with print option of particle size (area) and number. This analysis gave us the number of beads that were engulfed per cell in the bead engulfment assay. To be sure that beads outside the cells were not counted, stack images were checked, and snaps with least number of beads (3–5) lying outside the cells were chosen for further analysis so as to get cleaner, noise free and unbiased counting of beads engulfed by the cells. For each biological replicate, multiple cells (~10–15) were analysed using the aforementioned analysis per coverslip, for a total of 6 coverslips per biological replicate. Based on this analysis, the number of beads engulfed per cell for each biological replicate was averaged across all the cells counted for that biological replicate and graphically plotted as a single averaged value per biological replicate.

### 2.9 Gel‐based ABPP assays

Microglial cells were harvested by gently scraping in chilled PBS, lysed using probe sonicator (1 s on, 3 s off, 60% amplitude, 30 s) on ice, the membrane lysates were prepared as per protocols reported by us previously (Joshi et al., 2018; Kelkar et al., 2019; Singh et al., 2020) and the protein concentration in these membrane lysates was measured using the Pierce BCA kit as per manufacturer's instructions. The membrane lysates were diluted to a final concentration of 1 mg/mL for all the gel‐based activity‐based protein profiling (ABPP) assays. For competitive ABPP, these membrane lysates were first incubated with 10‐μM JJH350 or vehicle (DMSO) at 37°C for 45 min with constant shaking in a final volume of 100 μL. Thereafter, samples were incubated with fluorophosphonate‐rhodamine (FP‐Rh) probe (2 μM) at 37°C for 45 min with constant shaking recently reported by us (Kelkar et al., 2019;Kumar et al., 2021; Rajendran et al., 2020). Finally, the reactions were quenched by adding 40 μL of 4× SDS‐PAGE loading dye and boiling the samples for 10 min at 95°C. For Cu‐based alkyne azide click (CuAAC) chemistry‐based JJH350 labelling reactions, 100‐μL membrane lysates (at 1 mg/mL) were treated with 10‐μM JJH350 or vehicle (DMSO) at 37°C for 45 min with constant shaking. Thereafter, these treated membrane lysates were treated with click mixture for 45 min at 37°C with constant shaking. The click mixture (11 μL per reaction of 100 μL lysate) consisted of 6 μL of 1.7‐mM TBTA in 4:1 DMSO:*tert* butanol, 2 μL of 50‐mM copper sulphate, 2 μL of 50‐mM TCEP and 1 μL of rhodamine‐azide (10 mM in DMSO) as per standard protocols reported earlier (Niphakis et al., 2015; Ogasawara et al., 2019). The click chemistry reactions were quenched by adding 45 μL of 4× SDS‐PAGE loading dye. All the gel‐based ABPP samples were resolved on a 10% SDS‐PAGE gel and imaged for in‐gel fluorescence using the Syngene G‐Box Chemi‐XRQ gel documentation system as per standard protocols reported by us (Kumar et al., 2021; Rajendran et al., 2020).

### 2.10 Statistical analysis

All data in this paper were plotted and statistically analysed using the Prism 9 (version 9.1.0) software for macOS (GraphPad). All bar data in this paper are represented as the mean ± standard deviation (*SD*) from at least three independent biological replicates per experimental group. The Student's two‐tailed unpaired parametric *t* test was used to determine the statistical significance between different experimental groups, and a *p* value of <.05 was considered statistically significant in this study.

## 3 RESULTS

### 3.1 ABHD12‐null mice show heightened phagocytosis in the cerebellum

**FIGURE 2:**
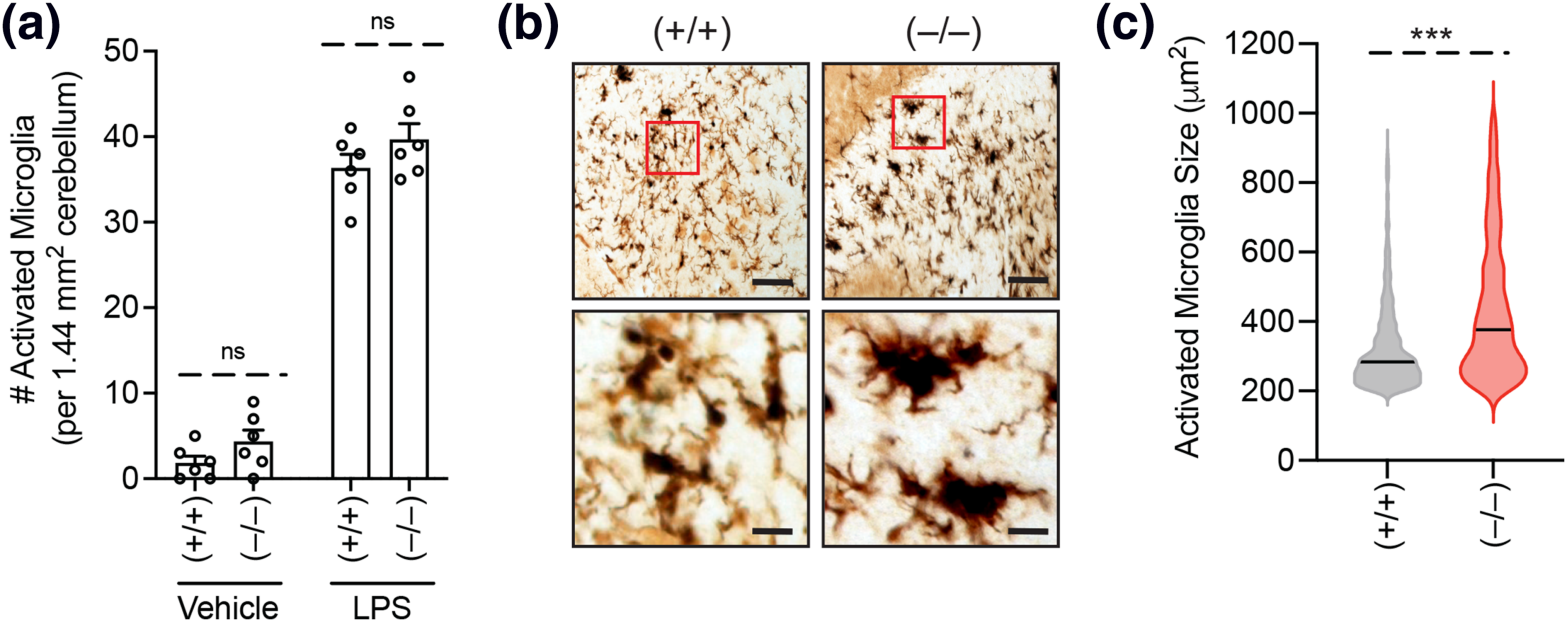
ABHD12‐null activated microglia in the arbor vitae region of the cerebellum have increased size. (a) Quantification of activated microglia (enlarged cells, >200 μm^2^ area) in the arbor vitae region of the cerebellum (per 1.44 mm^2^ area) of wild type (+/+) and ABHD12 knockout (−/−) mice following intraperitoneal injection of vehicle (1× PBS) or lipopolysaccharide (LPS) (10 mg/kg body weight) for 4 h. Data represent mean ± standard deviation from six independent experiments (biological replicates) per experimental group. (b) Representative Iba‐1 immunostaining image of the activated microglia in the arbor vitae region of the cerebellum of wild type (+/+) and ABHD12 knockout (−/−) mice following intraperitoneal injection of LPS (10 mg/kg body weight, 4 h), showing amoeboid morphology for activated microglia from the ABHD12 knockout mice. This experiment was done 6 times with reproducible results each time. Top images, scale bar = 250 μm; bottom zoomed images, scale bar = 10 μm. (c) Size of activated microglia in the arbor vitae region of the cerebellum of wild type (+/+) and ABHD12 knockout (−/−) mice following intraperitoneal injection of LPS (10 mg/kg body weight, 4 h), showing increased average size of activated microglia from the ABHD12 knockout mice. Violin plot data represent ensemble of sizes of 1085 individual activated microglia from the cerebellum from six independent experiments (biological replicates) per experimental group, and the black line in the plot represents average size of activated microglia for that experimental group. ***p < .001 versus (+/+) group by Student's two‐tailed unpaired parametric *t* test; ns = not statistically significant

The loss of ABHD12 activity in mice results in an accumulation of brain lyso‐PSs, particularly VLC lyso‐PSs in the cerebellum (Blankman et al., 2013; Khandelwal et al., 2021; Singh et al., 2020), that over time causes microglial activation, especially in the arbor vitae region closer to the deep nuclei in the cerebellum, given the localization of ABHD12 and the enrichment of the upstream PS lipase (major lyso‐PS biosynthetic enzyme) ABHD16A (Singh et al., 2020) to the cerebellum and therefore results in neuroinflammation (Blankman et al., 2013; Khandelwal et al., 2021). Following up on these seminal findings, we have recently shown from studies in the mammalian (mouse) brain that during inflammation caused by oxidative stress, ABHD12 also controls the levels of the potent pro‐apoptotic oxidized PSs, and its deletion results in increased brain oxidized PS levels (and likely manifests into neuroinflammation) (Kelkar et al., 2019). Given these recently published studies and the strong association of lyso‐PS/oxidized PS and ABHD12 with neuroinflammation (particularly in the cerebellum), we wanted to explore the effects of deletion of ABHD12 on microglial activation during an inflammatory stimulus. In our current studies, this inflammatory stimulus was provided by systemically injecting mice with lipopolysaccharide (LPS) (10 mg/kg body weight, intraperitoneally, 4 h), following which the brains of these treated mice were isolated, processed for IHC analysis, and the extent of microglial activation was assessed by counting the number of ionized calcium binding adaptor molecule (Iba‐1) stained cells having their soma size (area) > 200 μm^2^ per unit area in arbor vitae region of the cerebellum, using protocols recently reported by us (Khandelwal et al., 2021; Singh et al., 2020). From these studies, we found that while the inflammatory stimulus (LPS injection) markedly increased the number of activated microglia in the cerebellum (~10‐fold) relative to the vehicle (1× PBS) control, we did not notice any significant differences in total numbers between the two different genotypes, that is, the wild type (+/+) and ABHD12‐null (−/−) mice groups, for either the vehicle or LPS treatments (Figure 2a).

Interestingly, from this IHC experiment, we visually noticed that upon the inflammatory stimuli, activated microglia from both genotypes had different morphologies (Figure 2b). Upon closer inspection, we found that following the inflammatory stimulus, the activated microglia from wild type (+/+) mice had significantly more ramifications, whereas those from ABHD12‐null (−/−) mice adopted amoeboid shapes (Figure 2b), consistent with a heightened microglial activation state for the latter (Boche et al., 2013; Davis et al., 2017; Heindl et al., 2018). To ensure that there were no observational biases in our visual inspections, we performed these experiments blinded and computed the average size (area in μm^2^) of the activated microglia in the arbor vitae region close to the deep cerebellar nuclei in the brains of mice from both genotypes upon the inflammatory stimuli using the automated image analysing tool, ImageJ (Arena et al., 2017; Rueden et al., 2017). Here, we found that consistent with our visual observation of the amoeboid morphology, from six independent experiments (biological replicates), the average size of activated microglia was significantly larger in ABHD12 knockout mice (Figure S1). Next, we pooled all the data for size of the activated microglia for both genotypes from these six biological replicates (*N* = 1085) and found that the activated microglia from ABHD12‐null (−/−) mice had significantly greater average size (~434 μm^2^) compared with those from wild type (+/+) mice (~325 μm^2^) (Figure 2c).

**FIGURE 3:**
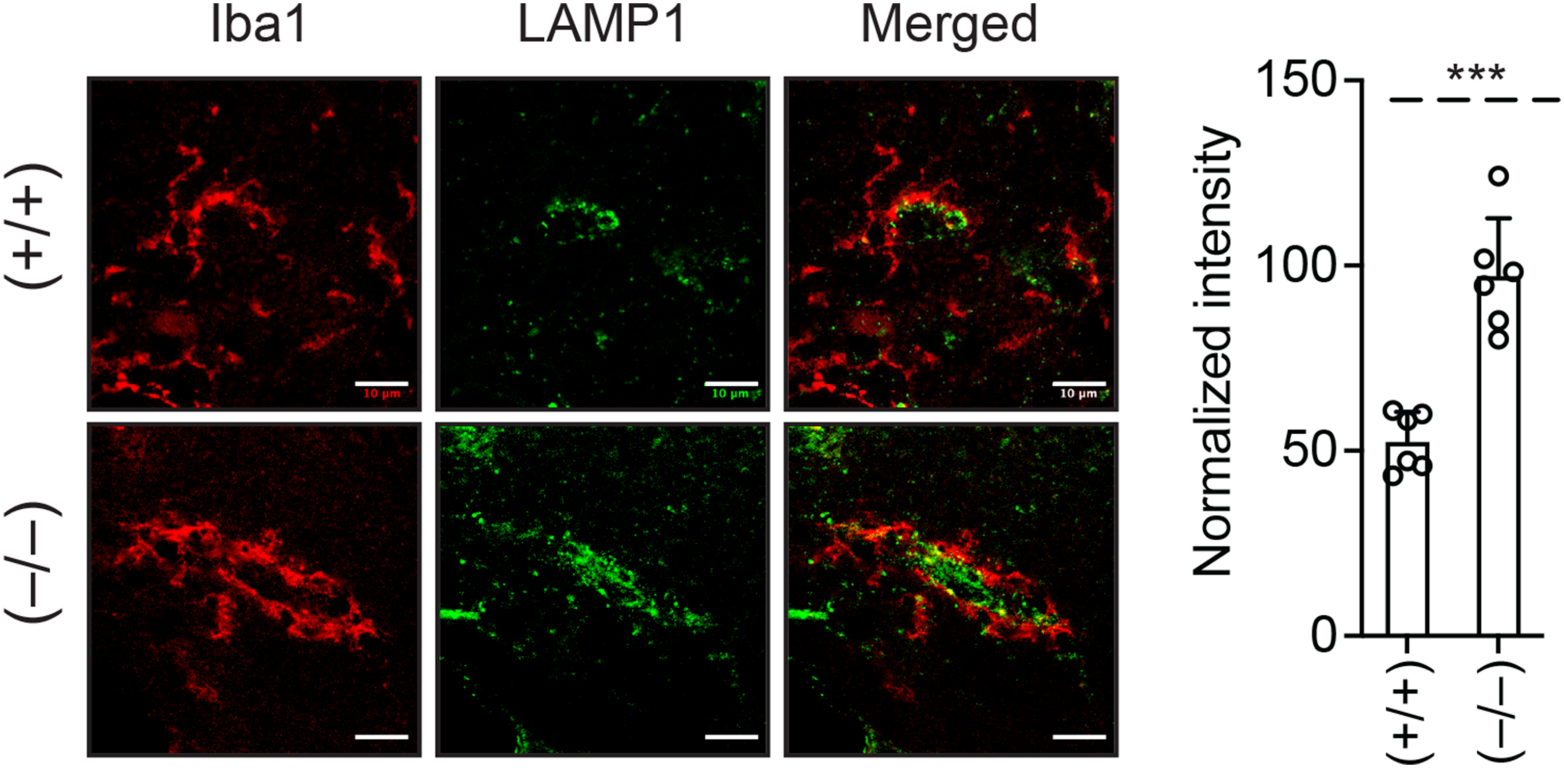
ABHD12‐null activated microglia in the cerebellum have increased LAMP1 expression. Representative immunofluorescence images and quantification of LAMP1 levels on activated microglia in the cerebellum of wild type (+/+) and ABHD12 knockout (−/−) mice following intraperitoneal injection of lipopolysaccharide (LPS) (10 mg/kg body weight, 4 hours), showing a ~2‐fold increase in the levels of LAMP1 on activated microglia from ABHD12 knockout mice. Data (bars) are represented as mean ± standard deviation from six independent experiments (biological replicates) per experimental group. Scale bar in the immunofluorescence images is 10 μm. ***p < .001 versus (+/+) group by Student's two‐tailed unpaired parametric *t* test

Heightened activation and an amoeboid morphology upon an inflammatory stimulus are both directly associated with increased phagocytosis activity by the microglia (Boche et al., 2013; Davis et al., 2017; Heindl et al., 2018). Previously, we have also shown that during the same inflammatory stimulus, there is an increased production of pro‐inflammatory cytokines (TNF‐α and IL‐6) in the brains of ABHD12 knockout mice relative to wild type controls, consistent with an increased activation status of the microglia of ABHD12‐null mice (Kelkar et al., 2019). Given this, we next wanted to investigate if this was indeed the case and if this was consistent with our immunohistological observations of activated microglia in the arbor vitae region of the cerebellum of ABHD12‐null (−/−) mice. Towards this, with the use of established IHC analysis (Khandelwal et al., 2021; Singh et al., 2020), we checked the activated microglia, for the presence of the lysosome‐associated membrane protein 1 (LAMP1), as this protein is present on late‐stage phagosomes (Pathak et al., 2018; Rai et al., 2016) and serves as a good indicator phagocytotic activity by macrophages and/or microglia. From this experiment, which were performed blinded, we found that upon an inflammatory stimulus, activated cerebellar microglia from ABHD12‐null (−/−) mice had significantly more LAMP1 levels (~2‐fold) relative to those from wild type (+/+) mice (Figure 3), consistent with an increased phagocytosis activity in the former, along with our previous finding (Kelkar et al., 2019), of the increased pro‐inflammatory cytokine production in the brains of ABHD12 knockout mice upon the same inflammatory stimulus.

### 3.2 Brain ABHD12 levels increase upon inflammatory stimuli

**FIGURE 4:**
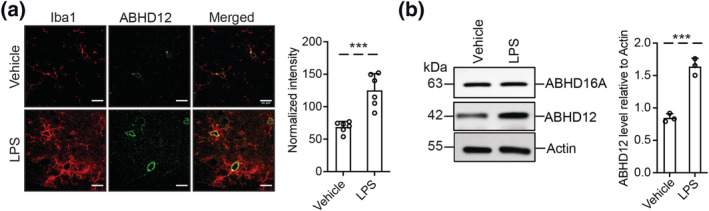
Brain ABHD12 levels increase upon an inflammatory stimulus. (a) Representative immunofluorescence images and quantification of ABHD12 levels on cerebellar microglia from wild type (+/+) mice, following intraperitoneal injection of vehicle (1× PBS) or LPS (10 mg/kg body weight, 4 h), showing a ~2‐fold increase in ABHD12 levels following lipopolysaccharide (LPS) treatment. Data (bars) are represented as mean ± standard deviation from six independent experiments (biological replicates) per experimental group. Scale bar in the immunofluorescence images is 10 μm. (b) Representative blot and quantification from western blot analysis of membrane lysates of the cerebellum from wild type (+/+) mice, following intraperitoneal injection of vehicle (1× PBS) or LPS (10 mg/kg body weight, 4 h), showing a ~2‐fold increase in ABHD12 levels following LPS treatment. The levels of the PS lipase ABHD16A in the cerebellum did not change following LPS treatment. Actin was used as a loading control for this experiment. This experiment was done three times (3 independent biological replicates) with reproducible results each time. For both (a) and (b), ***p < .001 versus (+/+) group by Student's two‐tailed unpaired parametric *t* test

Previous studies in primary macrophages have shown that upon an inflammatory stimulus (LPS treatment), the levels and, in turn, the lipase activity of ABHD12 significantly decrease (~2‐fold) with a concomitant increase in the concentrations of the lipid metabolites that it regulates, that is, the cellular oxidized PS lipids and secreted lyso‐PS lipids (Kamat et al., 2015; Kelkar et al., 2019; Singh et al., 2020). In addition to this, we have previously shown in a LPS‐based neuroinflammation paradigm that the concentration of lyso‐PSs in the brains of mice remains unchanged, whereas those of oxidized PS significantly increase and that ABHD12 regulates lyso‐PS levels during basal conditions (vehicle treatments) and oxidized PS levels during oxidative stress (LPS treatment) (Kelkar et al., 2019). Given this precedence, we wanted to know if the levels of ABHD12 in the wild type mammalian (mouse) brain indeed change during an inflammatory stimulus (LPS injection, 10 mg/kg body weight, intraperitoneally, 4 h). We decided to investigate this initially using IHC analysis of microglial cells in the arbor vitae region closer to the deep cerebellar nuclei, as previous studies have shown that primary microglia have high protein levels and hence increased activity of ABHD12 (Viader et al., 2016). Quite surprisingly, and opposite to the findings from primary macrophages (Kamat et al., 2015; Singh et al., 2020), we found that upon an inflammatory stimulus, the concentration of ABHD12 on activated microglia of the cerebellum actually significantly increased (~2‐fold) (Figure 4a). To further validate this finding, we decided to perform an immunoblotting (western blot) experiment on membrane lysates of the cerebellum, following an inflammatory stimulus. Here, we found consistent with the IHC analysis (Figure 4a), that relative to the vehicle (PBS) control, the levels of ABHD12 in the cerebellum were indeed significantly elevated (~2‐fold) upon an inflammatory stimulus (LPS injection, 10 mg/kg body weight, intraperitoneally, 4 h) (Figure 4b). In this immunoblotting experiment, the cytoskeleton protein β‐actin was used as an equal loading control (Figure 4b). In primary macrophages, it has also been shown that the protein levels and therefore the lipase activity of the major lyso‐PS biosynthetic enzyme (PS lipase) ABHD16A significantly increase upon an inflammatory stimulus (Kamat et al., 2015; Singh et al., 2020), and hence, we decided to check if the levels of this PS lipase were altered in our immunoblotting experiment on membrane lysates of the cerebellum. Interestingly, we did not find any changes in levels of the PS lipase ABHD16A in the cerebellum upon an inflammatory stimulus (Figure 4b), consistent with our previous findings that show that the lyso‐PS levels remain unchanged in a LPS‐based neuroinflammation paradigm (Kelkar et al., 2019).

### 3.3 Disruption of ABHD12 increases phagocytosis by microglial cells

**FIGURE 5:**
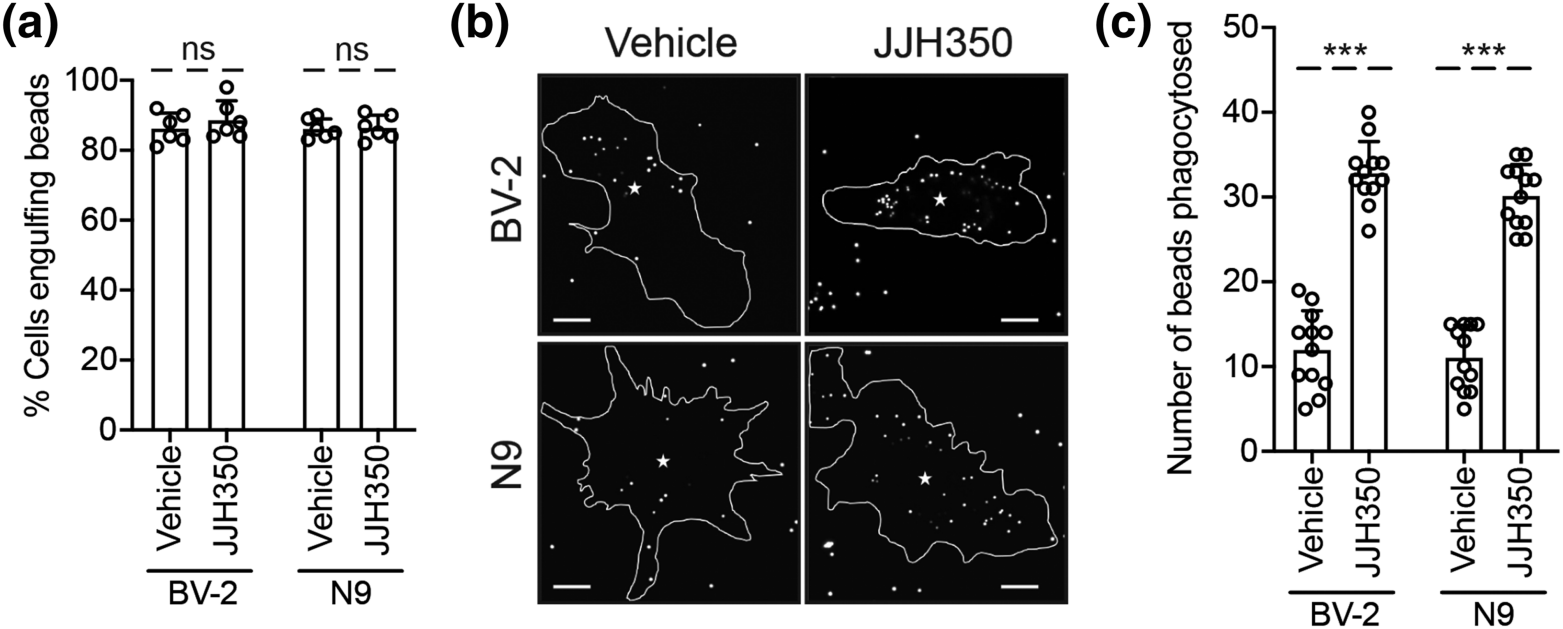
Pharmacological inhibition of ABHD12 results in increased phagocytosis by microglial cells in a fluorescent bead uptake assay. (a) Quantification of the total percentage of microglial cells (BV‐2 and N9) engulfing fluorescent beads following vehicle (DMSO) or JJH350 (10 μM, 6 h) treatment, showing no significant differences between the two experimental groups. Data (bars) are represented as mean ± standard deviation from six independent experiments (biological replicates) per experimental group. (b) Representative microscopy images of microglial cells (BV‐2 and N9) engulfing fluorescent beads following vehicle (DMSO) or JJH350 (10 μM, 6 h) treatment, showing increased phagocytosis of fluorescent beads in the JJH350‐treated microglial cells. Scale bar in the microscopy images is 10 μm, and (*) represents centroid of the nucleus. (c) The number of fluorescent beads phagocytosed by microglial cells (BV‐2 and N9) following vehicle (DMSO) or JJH350 (10 μM, 6 h) treatment, showing a significant increase of number of phagocytosed fluorescent beads by the JJH350‐treated microglial cells (BV‐2 and N9). Data (bars) are represented as mean ± standard deviation from 12 independent experiments (biological replicates) per experimental group. ***p < .001 versus vehicle group by Student's two‐tailed unpaired parametric *t* test; ns = not statistically significant

That upon an inflammatory stimulus, the microglia in ABHD12‐null mice have heightened activation status, an amoeboid morphology, and higher levels of the phagocytic marker LAMP1 strongly point to a role of ABHD12 in modulating phagocytosis (Figures 2 and 3). Additionally, the increase in cerebellar ABHD12 protein levels upon an inflammatory stimulus further suggests that this lipase controls levels of the pro‐apoptotic oxidized PS in the mammalian brain during oxidative stress and possibly tempers heightened phagocytosis during such physiological conditions (Figure 4). Given these findings, we next wanted to assess, whether the pharmacological inhibition and/or genetic deletion of ABHD12 in microglial cells had any effect on the rate of phagocytosis or phagocytic activity in these innate immune cells. For these experiments, we chose the well‐studied BV‐2 and N9 immortalized microglial cell lines and first confirmed that these microglial cells indeed express ABHD12 by western blotting (Figure S2). Next, we also confirmed by established competitive gel‐based ABPP assays (Kelkar et al., 2019; Kulkarni et al., 2019) that the *N*‐hydroxyhydantoin‐carbamate JJH350 (10 μM, 6 h), a recently reported ABHD12 selective covalent inhibitor (Ogasawara et al., 2019), indeed reacted with, and covalently inhibited ABHD12 in an irreversible manner in both the BV‐2 and N9 microglial cells, and was therefore used for the pharmacological inhibition of ABHD12 in all our subsequent assays in these cell lines (Figure S3). Next, to determine the effect of ABHD12 inhibition on phagocytosis, we treated the BV‐2 or N9 cells with vehicle (DMSO) or JJH350 (10 μM, 6 h), following which they were incubated with fluorescent latex beads (0.5 μm, cell to bead ratio = 1:200 for 30 min), and the number of beads that were engulfed by the microglial cells was visualized by fluorescence microscopy using established protocols (Rajendran et al., 2020; Singh et al., 2020). It is known that these fluorescent latex beads act as antigens and are known to trigger oxidative stress response due to activation of inflammatory and/or phagocytic pathways in phagocytes (Luo et al., 2016; Pilat et al., 2013). From these bead‐engulfment (phagocytosis) assays, we found that majority of the microglial cells (~90% of the total cells) engulfed the fluorescent latex beads, and for either treatment (vehicle or JJH350), the percentage of total cells engulfing the fluorescent latex beads for both the BV‐2 and N9 microglia was almost identical (Figure 5a). On closer visual inspection of the microscopy images, we found that the JJH350 treated microglia engulfed markedly more fluorescent beads than the vehicle treated microglia for both the BV‐2 and N9 microglial cells (Figure 5b). Next, upon counting the number of fluorescent latex beads engulfed per phagocytosing microglial cell, we found that the JJH350 treated microglia engulfed ~3 times more fluorescent latex beads than the corresponding vehicle treated microglia (average fluorescent latex beads engulfed per phagocytosing cell for BV‐2 microglia: vehicle = 12, JJH350 = 33; N9 microglia: vehicle = 11, JJH350 = 30) (Figure 5c).

**FIGURE 6:**
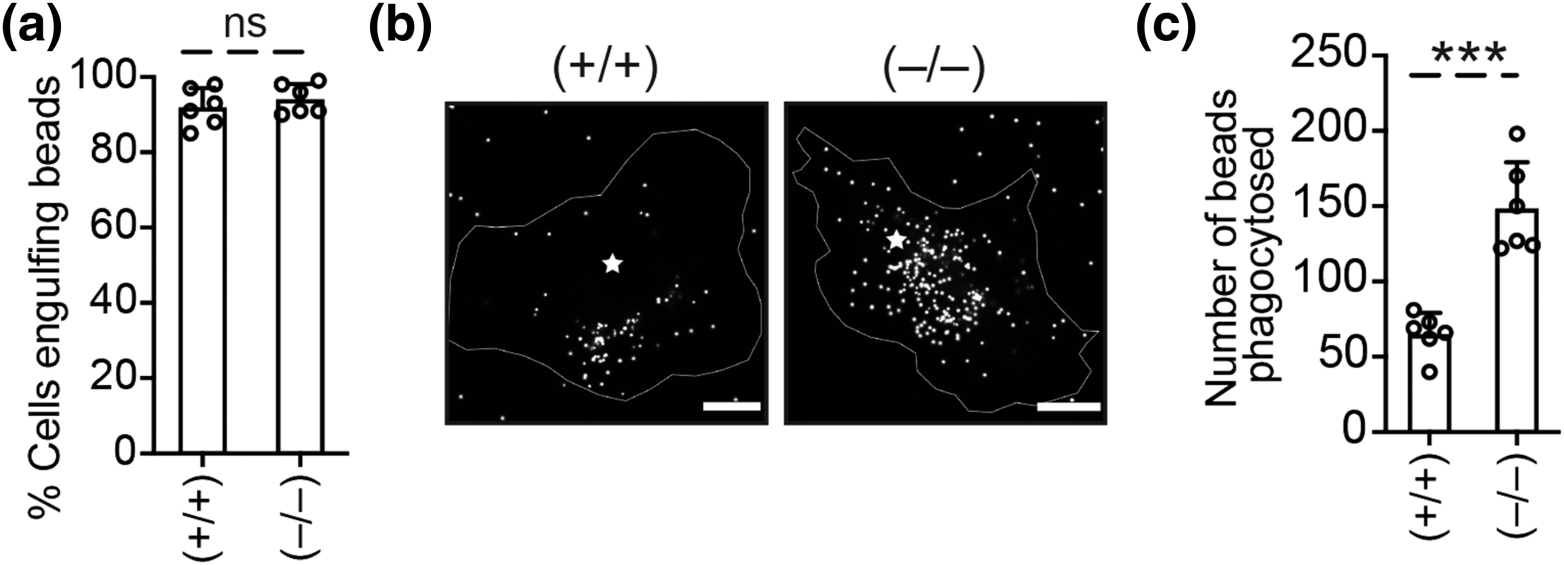
Deletion of ABHD12 in primary microglial cells results in increased phagocytosis in a fluorescent bead uptake assay. (a) Quantification of the total percentage of primary microglial cells obtained from wild type (+/+) and ABHD12 knockout (−/−) mice engulfing fluorescent beads, showing no significant differences between the two genotypes. Data (bars) are represented as mean ± standard deviation from six independent experiments (biological replicates) per genotype. (b) Representative microscopy images of primary microglial cells obtained from wild type (+/+) and ABHD12 knockout (−/−) mice, engulfing fluorescent latex beads, showing increased phagocytosis activity towards engulfing the fluorescent latex beads in the ABHD12‐null primary microglial cells. Scale bar in the microscopy images is 10 μm, and (*) represents centroid of the nucleus. (C) The number of fluorescent beads phagocytosed by primary microglial cells obtained from wild type (+/+) and ABHD12 knockout (−/−) mice, showing a significant increase of number of phagocytosed fluorescent latex beads by ABHD12‐null primary microglial cells. Data (bars) are represented as mean ± standard deviation from six independent experiments (biological replicates) per genotype. ***p < .001 versus vehicle group by Student's two‐tailed unpaired parametric *t* test; ns = not statistically significant

To complement these pharmacological studies, with the use of established protocols (Viader et al., 2016), we harvested primary microglial cells from wild type (+/+) and ABHD12 knockout (−/−) mice and performed the same fluorescent latex bead engulfment assays to determine, whether the genetic deletion of ABHD12 also has any effect on the phagocytic activity of primary microglial cells. In these cellular phagocytosis assays, we found that the majority of the primary microglial cells (~90% of the total cells) engulfed the fluorescent latex beads, and in terms of the percentage of total primary microglial cells engulfing these fluorescent beads, we found that for both the genotypes these numbers were almost identical (Figure 6a). Upon closer visual inspection of the microscopy images from these cellular phagocytosis assays, we found that the ABHD12‐null (−/−) primary microglial cells engulfed significantly more fluorescent beads compared with wild type (+/+) primary microglial cells (Figure 6b). On quantifying the number of fluorescent latex beads engulfed per phagocytosing primary microglial cell, we found that the ABHD12‐null (−/−) primary microglial cells engulfed ~2 times more fluorescent beads than the corresponding wild type (+/+) primary microglia (average fluorescent latex beads engulfed per phagocytosing cell by primary microglia: wild type (+/+) = 65, and ABHD12‐null (−/−) = 150) (Figure 6c). Interestingly, we also performed these cellular phagocytosis assays in primary thioglycollate‐elicited peritoneal macrophages derived from wild type (+/+) or ABHD12‐null (−/−) mice (Kelkar et al., 2019; Singh et al., 2020) and found results similar to those observed in the microglial cells that the loss of ABHD12 activity in macrophages resulted in significantly more (~3 times) phagocytosis of fluorescent latex beads (Figure S4).

## 4 DISCUSSION

Little over a decade ago, deleterious null mutations in the gene that encodes the integral membrane‐associated lipase ABHD12 were identified as the cause for the early onset human neurological disorder PHARC, which is clinically represented in human subjects as polymodal sensory and motor defects caused by peripheral neuropathy, coupled with an early onset of cataract (and/or blindness), and hearing loss. Since this initial discovery, several studies have been published that describe the biochemical activities of ABHD12 and the lipid signalling pathways that this disease‐associated lipase regulates in mammals, including humans. In a seminal study by Cravatt and co‐workers, they generated the ABHD12 knockout mouse and showed this rodent model serves as an excellent preclinical surrogate to study various aspects of the pathophysiology of PHARC. In the same study, it was chronologically shown that the loss of the ABHD12 lipase activity first results in the accumulation of the bioactive lyso‐PSs in the brain (Figure 1) that causes deregulated (lyso‐PS‐mediated) signalling over time, particularly in the cerebellum (given the localization of ABHD12 and the upstream lyso‐PS biosynthetic enzyme ABHD16A in the cerebellum) of ABHD12 knockout mice. This aberrant lyso‐PS signalling is then succeeded by microglial activation in the arbor vitae region of the cerebellum and thereby (neuro)inflammation, which likely manifests into behavioural (motor and auditory) defects in the ABHD12 knockout mice, mirroring those seen in human PHARC subjects (Figure 1). Following up on these findings, we have recently shown that, by its lipase activity, ABHD12 also controls the physiological concentrations of the potent pro‐apoptotic oxidized PS lipids (Figure 1) and significantly tempers inflammatory responses under conditions of oxidative stress, particularly in the mammalian brain and macrophages. Interestingly, both the lyso‐PS and oxidized PS lipids have had a strong association with the activation of macrophage cells, and phagocytosis, and yet despite its biomedical importance, the effect that the loss of ABHD12 activity has on phagocytosis and the correlation of phagocytosis to the pathological phenotypes associated with PHARC has not been thoroughly investigated or reported to the best of our knowledge.

Given the role of ABHD12 in modulating levels of the immunomodulatory lyso‐PSs and pro‐apoptotic oxidized PSs in macrophages, we decided to investigate whether the loss of its lipase activity has any effect on phagocytosis. Towards this, we first challenged wild type and ABHD12 knockout mice with an inflammatory stimulus (systemic LPS injection) and checked the extent of neuroinflammation in the arbor vitae region of the cerebellum, by counting the number of activated microglia using established IHC analysis. We found in this anatomical region of the cerebellum that while the total number of activated microglia between the two genotypes did not change, those from ABHD12 knockout mice adopted an amoeboid morphology and had a greater soma size (Figure 2), with a concomitant increase in the phagocytic marker protein LAMP1 (Figure 3), all together consistent with a heightened phagocytosis activity in ABHD12‐null cerebellar microglia. We have previously shown that the pro‐apoptotic oxidized PS lipids accumulate in the brains of ABHD12 knockout mice during conditions of oxidative stress (e.g., during systemic LPS injection). Interestingly, and contrary to findings in macrophages, here, we show that during such oxidative stress conditions, in the wild type mice brains, the levels of ABHD12 increase nearly twofold (Figure 4). We think this is an innate anticipatory mechanism in the mammalian brain, where by increasing the levels of ABHD12 in response to an inflammatory stimulus, excess oxidized PSs produced during oxidative stress in the brain (particularly in the cerebellum) are efficiently metabolized by the oxidized PS lipase activity of ABHD12, and therefore, the apoptosis and/or phagocytosis due to the accumulation of the oxidized PS lipids in neurons or other brain resident glial cells is prevented. Next, we wanted to directly assess in cultured microglial cells, if the loss of ABHD12 activity indeed resulted in increased phagocytosis and decided to address this directly using an established fluorescent latex bead‐uptake assay using complementary pharmacological and genetic approaches. We found from this assay in two immortalized microglial cell lines (BV‐2 and N9) that the pharmacological inhibition of ABHD12 activity resulted in significantly more phagocytosis activity and hence an increased uptake of fluorescent latex beads in both cell lines (Figure 5). We also performed the same bead‐uptake assay in primary microglia cells that were obtained from wild type (+/+) or ABHD12‐null (−/−) mice and found that the deletion of ABHD12 and loss of its lipase activity in primary microglial cells result in significantly more uptake of fluorescent latex beads via phagocytosis (Figure 6). Interestingly, we found that the loss of the ABHD12 activity also resulted in similar increased phagocytosis activity in primary peritoneal macrophages (Figure S4). Taken together, all our data provide compelling evidence both in vivo and by in vitro biochemical experiments that the loss of ABHD12 activity indeed causes heightened phagocytosis activity in microglial (and macrophage) cells.

**FIGURE 7:**
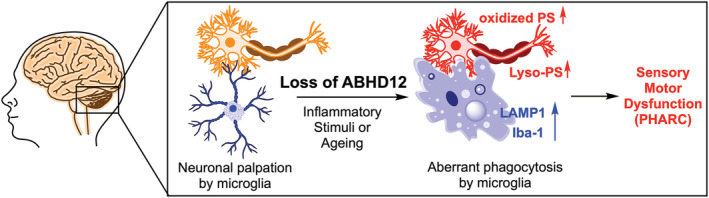
A model summarizing the increased phagocytosis observed in the cerebellar microglial cells in ABHD12 knockout mice

We think that the association of loss of ABHD12 activity with increased phagocytosis activity has tremendous implications in the context of the pathophysiology of PHARC and proposes a model based on the findings in this paper, for explaining this (Figure 7). We have recently shown why the cerebellum is the most atrophic region of the brain in human PHARC subjects given the distinct localization of ABHD12 and ABHD16A to the Purkinje and Granular neurons respectively (Singh et al., 2020). During normal conditions, the microglia are constantly palpating various neurons present in the cerebellum, and phagocytosis by these microglia is at a minimal, given that most cerebellar neurons are healthy, and signalling events are fairly homeostatic and well‐regulated at this point. In human PHARC subjects, we speculate that the loss of ABHD12 activity results in the buildup lyso‐PS (even during basal conditions because of the sustained PS lipase activity of ABHD16A in the cerebellum) (Blankman et al., 2013; Kamat et al., 2015; Singh et al., 2020) and oxidized PS (during some inflammatory stimuli, oxidative stress) (Kelkar et al., 2019) in the cerebellum, presumably from Purkinje neurons and cerebellar microglial cells that highly express ABHD12 in this brain region. Together, the increased concentrations of the immunomodulatory lyso‐PS and pro‐apoptotic oxidized PS lipids because of loss of ABHD12 activity further activate cerebellar microglia and initiate a heightened phagocytosis response that we posit leads to the continued neuronal damage in the cerebellum. This damage to cerebellar (likely Purkinje) neurons eventually manifests into the sensory and motor defects observed in human PHARC subjects. We also speculate that the heightened phagocytosis activity by microglial cells due to the loss of ABHD12, over time, also causes the cerebellar atrophy observed in human PHARC subjects.

Moving ahead, it will be interesting to mechanistically understand and delineate how the flux and interplay between lyso‐PSs and oxidized PSs intricately regulated by ABHD12 in the brain, particularly in the cerebellar microglial cells and neurons, influence the various phagocytosis pathways that culminate into neuroinflammation and lead to the behavioural defects associated with PHARC. Towards understanding this, it will be very useful to generate much needed brain cell‐specific ABHD12 knockout mice models (e.g., deletion of ABHD12 specifically in neurons or microglia) to map this interplay in the central nervous system and dissect out contribution of different cells in the brain in causing the pathology observed in PHARC. Currently, very little (if at all anything) is known of the putative receptors for both lyso‐PSs and oxidized PSs in microglial cells, and the mammalian brain in general, and therefore identifying and annotating such as‐of‐yet unknown receptors, will add tremendous value to our understanding of phagocytosis pathways in the brain associated with both these signalling lipids. We have recently shown that VLC lyso‐PSs, that accumulate most in the brains of ABHD12 knockout mice, potently activate macrophages via a TLR2‐dependent pathway (Khandelwal et al., 2021), and it would be interesting to mechanistically investigate the VLC lyso‐PS–TLR2 axis in the context of phagocytosis and neuroinflammation observed in PHARC. Finally, on a same note, from a translational perspective, it will also be interesting to assess if the microglial activation associated with ABHD12 deletion in the murine model of PHARC can be rectified by treatment with pharmacological agents such as the neuroprotective antibiotic minocycline, and if so, this can be translated into a therapy for treating the neuroinflammation observed in human PHARC subjects.

## AUTHOR CONTRIBUTIONS

S.S. performed all the experiments. S.S. and S.S.K. conceived the project, analysed all the data and wrote the manuscript. S.S.K. acquired funding and supervised this study.

## CONFLICT OF INTEREST

The authors declare no competing financial interests.

### PEER REVIEW

The peer review history for this article is available at https://publons.com/publon/10.1111/ejn.15516.

## Supporting information


**Figure S1.** **Size of activated cerebellar microglial cells per biological replicate.** The size of activated microglia in the arbor vitae region of the cerebellum of wild type (+/+) and ABHD12 knockout (−/−) mice following intraperitoneal injection of LPS (10 mg/kg body weight, 4 hours), showing increased average size of activated microglia from the ABHD12 knockout mice in every biological replicate tested. The “n” in parenthesis represents number of activated cerebellar microglial cells quantified per biological replicate. ***p < 0.001 versus (+/+) group by Student's two‐tailed unpaired parametric *t*‐test.
**Figure S2. ABHD12 is expressed in the BV‐2 and N9 microglial cell lines.** Western blot analysis showing the expression of ABHD12 in the BV‐2 and N9 microglial cells. In this experiment, Iba‐1 and GAPDH were used as loading controls. This western blot experiment was done two times (two separate biological replicates) with reproducible results both times.
**Figure S3. Competitive gel‐based activity based protein profiling (ABPP) assays showing inhibition of ABHD12 by JJH350 in microglial cells**. (**A**) Competitive gel‐based ABPP assays on membrane lysates of BV‐2 and N9 microglial cells treated with DMSO or JJH350 (10 μM, 6 hours) using the serine‐hydrolase directed FP‐rhodamine probe (2 μM, 45 mins) showing loss of ABHD12 following JJH350 treatment. (**B**) Click reactions on membrane lysates of BV‐2 and N9 microglial cells treated with DMSO or JJH350 (10 μM, 6 hours) showing that JJH350 binds selectively to ABHD12 as per literature reports. Both experiments were done thrice (biological replicates) with reproducible results each time. The red double asterisk on all the ABPP gels correspond to the ABHD12 activity band.
**Figure S4. Loss of ABHD12 results in increased phagocytosis by primary peritoneal macrophages in a fluorescent latex bead uptake assay**. (**A**) Quantification of the total percentage of primary thioglycollate‐elicited peritoneal macrophages engulfing fluorescent latex beads following vehicle (DMSO) or JJH350 (10 μM, 6 hours) treatment, showing no significant differences between the two experimental groups. Data (bar plots) is represented as mean ± standard deviation from six independent experiments (biological replicates) per experimental group. (**B**) Representative microscopy images of primary thioglycollate‐elicited peritoneal macrophages engulfing fluorescent beads following vehicle (DMSO) or JJH350 (10 μM, 6 hours) treatment, showing increased phagocytosis of fluorescent beads in the JJH350‐treated primary thioglycollate‐elicited peritoneal macrophages. Scale bar in the microscopy images is 10 μm and (*) represents centroid of the nucleus. (**C**) The number of fluorescent beads phagocytosed by primary thioglycollate‐elicited peritoneal macrophages following vehicle (DMSO) or JJH350 (10 μM, 6 hours) treatment, showing a significant increase of number of phagocytosed fluorescent latex beads by the JJH350‐treated primary thioglycollate‐elicited peritoneal macrophages. Data (bars) is represented as mean ± standard deviation from twelve independent experiments (biological replicates) per experimental group. ***p < 0.001 versus vehicle group by Student's two‐tailed unpaired parametric *t*‐test.

## Data Availability

All data associated with this study are available within the paper or its associated supporting information or can be made available by the corresponding author upon reasonable request.

## References

[ejn15516-bib-0001] Arena, E. T. , Rueden, C. T. , Hiner, M. C. , Wang, S. , Yuan, M. , & Eliceiri, K. W. (2017). Quantitating the cell: Turning images into numbers with ImageJ. Wiley Interdisciplinary Reviews: Developmental Biology, 6(2), e260. 10.1002/wdev.260 27911038

[ejn15516-bib-0002] Blankman, J. L. , Long, J. Z. , Trauger, S. A. , Siuzdak, G. , & Cravatt, B. F. (2013). ABHD12 controls brain lysophosphatidylserine pathways that are deregulated in a murine model of the neurodegenerative disease PHARC. Proceedings of the National Academy of Sciences of the United States of America, 110(4), 1500–1505. 10.1073/pnas.1217121110 23297193 PMC3557017

[ejn15516-bib-0003] Boche, D. , Perry, V. H. , & Nicoll, J. A. (2013). Review: Activation patterns of microglia and their identification in the human brain. Neuropathology and Applied Neurobiology, 39(1), 3–18. 10.1111/nan.12011 23252647

[ejn15516-bib-0004] Davis, B. M. , Salinas‐Navarro, M. , Cordeiro, M. F. , Moons, L. , & de Groef, L. (2017). Characterizing microglia activation: A spatial statistics approach to maximize information extraction. Scientific Reports, 7(1), 1576. 10.1038/s41598-017-01747-8 28484229 PMC5431479

[ejn15516-bib-0005] Doran, A. C. , Yurdagul, A. Jr. , & Tabas, I. (2020). Efferocytosis in health and disease. Nature Reviews. Immunology, 20(4), 254–267. 10.1038/s41577-019-0240-6 PMC7667664 31822793

[ejn15516-bib-0006] Elliott, M. R. , Koster, K. M. , & Murphy, P. S. (2017). Efferocytosis signaling in the regulation of macrophage inflammatory responses. Journal of Immunology, 198(4), 1387–1394. 10.4049/jimmunol.1601520 PMC5301545 28167649

[ejn15516-bib-0007] Fiskerstrand, T. , H'Mida‐Ben Brahim, D. , Johansson, S. , M'Zahem, A. , Haukanes, B. I. , Drouot, N. , Zimmermann, J. , Cole, A. J. , Vedeler, C. , Bredrup, C. , Assoum, M. , Tazir, M. , Klockgether, T. , Hamri, A. , Steen, V. M. , Boman, H. , Bindoff, L. A. , Koenig, M. , & Knappskog, P. M. (2010). Mutations in ABHD12 cause the neurodegenerative disease PHARC: An inborn error of endocannabinoid metabolism. American Journal of Human Genetics, 87(3), 410–417. http://www.ncbi.nlm.nih.gov/pubmed/20797687 20797687 10.1016/j.ajhg.2010.08.002 PMC2933347

[ejn15516-bib-0008] Fiskerstrand, T. , Knappskog, P. , Majewski, J. , Wanders, R. J. , Boman, H. , & Bindoff, L. A. (2009). A novel Refsum‐like disorder that maps to chromosome 20. Neurology, 72(1), 20–27. Retrieved from. http://www.neurology.org/content/72/1/20.abstract, 10.1212/01.wnl.0000333664.90605.23 19005174

[ejn15516-bib-0009] Flannagan, R. S. , Jaumouille, V. , & Grinstein, S. (2012). The cell biology of phagocytosis. Annual Review of Pathology, 7, 61–98. 10.1146/annurev-pathol-011811-132445 21910624

[ejn15516-bib-0010] Frasch, S. C. , Berry, K. Z. , Fernandez‐Boyanapalli, R. , Jin, H. S. , Leslie, C. , Henson, P. M. , Murphy, R. C. , & Bratton, D. L. (2008). NADPH oxidase‐dependent generation of lysophosphatidylserine enhances clearance of activated and dying neutrophils via G2A. The Journal of Biological Chemistry, 283(48), 33736–33749. 10.1074/jbc.M807047200 18824544 PMC2586279

[ejn15516-bib-0011] Frasch, S. C. , & Bratton, D. L. (2012). Emerging roles for lysophosphatidylserine in resolution of inflammation. Progress in Lipid Research, 51(3), 199–207. http://www.ncbi.nlm.nih.gov/pubmed/22465125 22465125 10.1016/j.plipres.2012.03.001 PMC3365616

[ejn15516-bib-0012] Greenberg, M. E. , Li, X. M. , Gugiu, B. G. , Gu, X. , Qin, J. , Salomon, R. G. , & Hazen, S. L. (2008). The lipid whisker model of the structure of oxidized cell membranes. The Journal of Biological Chemistry, 283(4), 2385–2396. 10.1074/jbc.M707348200 18045864

[ejn15516-bib-0013] Greenberg, M. E. , Sun, M. , Zhang, R. , Febbraio, M. , Silverstein, R. , & Hazen, S. L. (2006). Oxidized phosphatidylserine‐CD36 interactions play an essential role in macrophage‐dependent phagocytosis of apoptotic cells. The Journal of Experimental Medicine, 203(12), 2613–2625. 10.1084/jem.20060370 17101731 PMC2118161

[ejn15516-bib-0014] Hazen, S. L. (2008). Oxidized phospholipids as endogenous pattern recognition ligands in innate immunity. The Journal of Biological Chemistry, 283, 15527–15531. 10.1074/jbc.R700054200 18285328 PMC2414290

[ejn15516-bib-0015] Heindl, S. , Gesierich, B. , Benakis, C. , Llovera, G. , Duering, M. , & Liesz, A. (2018). Automated morphological analysis of microglia after stroke. Frontiers in Cellular Neuroscience, 12, 106. 10.3389/fncel.2018.00106 29725290 PMC5917008

[ejn15516-bib-0016] Joshi, A. , Shaikh, M. , Singh, S. , Rajendran, A. , Mhetre, A. , & Kamat, S. S. (2018). Biochemical characterization of the PHARC‐associated serine hydrolase ABHD12 reveals its preference for very‐long‐chain lipids. Journal of Biological Chemistry, 293(44), 16953–16963. 10.1074/jbc.RA118.005640 30237167 PMC6217928

[ejn15516-bib-0017] Kagan, V. E. , Gleiss, B. , Tyurina, Y. Y. , Tyurin, V. A. , Elenstrom‐Magnusson, C. , Liu, S. X. , Serinkan, F. B. , Arroyo, A. , Chandra, J. , Orrenius, S. , & Fadeel, B. (2002). A role for oxidative stress in apoptosis: Oxidation and externalization of phosphatidylserine is required for macrophage clearance of cells undergoing Fas‐mediated apoptosis. Journal of Immunology, 169(1), 487–499. https://www.ncbi.nlm.nih.gov/pubmed/12077280 10.4049/jimmunol.169.1.487 12077280

[ejn15516-bib-0018] Kamat, S. S. , Camara, K. , Parsons, W. H. , Chen, D. H. , Dix, M. M. , Bird, T. D. , Howell, A. R. , & Cravatt, B. F. (2015). Immunomodulatory lysophosphatidylserines are regulated by ABHD16A and ABHD12 interplay. Nature Chemical Biology, 11(2), 164–171. 10.1038/nchembio.1721 25580854 PMC4301979

[ejn15516-bib-0019] Kelkar, D. S. , Ravikumar, G. , Mehendale, N. , Singh, S. , Joshi, A. , Sharma, A. K. , Mhetre, A. , Rajendran, A. , Chakrapani, H. , & Kamat, S. S. (2019). A chemical‐genetic screen identifies ABHD12 as an oxidized‐phosphatidylserine lipase. Nature Chemical Biology, 15(2), 169–178. 10.1038/s41589-018-0195-0 30643283 PMC6420073

[ejn15516-bib-0020] Khandelwal, N. , Shaikh, M. , Mhetre, A. , Singh, S. , Sajeevan, T. , Joshi, A. , Balaji, K. N. , Chakrapani, H. , & Kamat, S. S. (2021). Fatty acid chain length drives lysophosphatidylserine‐dependent immunological outputs. Cell Chemical Biology, 28(8), 1169–1179. 10.1016/j.chembiol.2021.01.008 33571455 PMC7611549

[ejn15516-bib-0021] Kinchen, J. M. , & Ravichandran, K. S. (2008). Phagosome maturation: Going through the acid test. Nature Reviews. Molecular Cell Biology, 9(10), 781–795. 10.1038/nrm2515 18813294 PMC2908392

[ejn15516-bib-0022] Kulkarni, A. , Soni, I. , Kelkar, D. S. , Dharmaraja, A. T. , Sankar, R. K. , Beniwal, G. , Rajendran, A. , Tamhankar, S. , Chopra, S. , Kamat, S. S. , & Chakrapani, H. (2019). Chemoproteomics of an indole‐based Quinone epoxide identifies Druggable vulnerabilities in vancomycin‐resistant Staphylococcus aureus. Journal of Medicinal Chemistry, 62(14), 6785–6795. 10.1021/acs.jmedchem.9b00774 31241934 PMC6660313

[ejn15516-bib-0023] Kumar, K. , Mhetre, A. , Ratnaparkhi, G. S. , & Kamat, S. S. (2021). A superfamily‐wide activity atlas of serine hydrolases in Drosophila melanogaster. Biochemistry, 60(16), 1312–1324. 10.1021/acs.biochem.1c00171 33827210 PMC7610703

[ejn15516-bib-0024] Levin, R. , Grinstein, S. , & Canton, J. (2016). The life cycle of phagosomes: Formation, maturation, and resolution. Immunological Reviews, 273(1), 156–179. 10.1111/imr.12439 27558334

[ejn15516-bib-0025] Luo, B. , Wang, J. , Liu, Z. , Shen, Z. , Shi, R. , Liu, Y. Q. , Liu, Y. , Jiang, M. , Wu, Y. , & Zhang, Z. (2016). Phagocyte respiratory burst activates macrophage erythropoietin signalling to promote acute inflammation resolution. Nature Communications, 7, 12177. 10.1038/ncomms12177 PMC4942576 27397585

[ejn15516-bib-0026] Martin, C. J. , Peters, K. N. , & Behar, S. M. (2014). Macrophages clean up: Efferocytosis and microbial control. Current Opinion in Microbiology, 17, 17–23. 10.1016/j.mib.2013.10.007 24581688 PMC3942671

[ejn15516-bib-0027] Matsura, T. , Togawa, A. , Kai, M. , Nishida, T. , Nakada, J. , Ishibe, Y. , Kojo, S. , Yamamoto, Y. , & Yamada, K. (2005). The presence of oxidized phosphatidylserine on Fas‐mediated apoptotic cell surface. Biochimica et Biophysica Acta, 1736(3), 181–188. 10.1016/j.bbalip.2005.08.011 16168707

[ejn15516-bib-0028] Murray, P. J. , & Wynn, T. A. (2011). Protective and pathogenic functions of macrophage subsets. Nature Reviews. Immunology, 11(11), 723–737. 10.1038/nri3073 PMC3422549 21997792

[ejn15516-bib-0029] Niphakis, M. J. , Lum, K. M. , Cognetta, A. B. 3rd , Correia, B. E. , Ichu, T. A. , Olucha, J. , Brown, S. J. , Kundu, S. , Piscitelli, F. , Rosen, H. , & Cravatt, B. F. (2015). A global map of lipid‐binding proteins and their Ligandability in cells. Cell, 161(7), 1668–1680. 10.1016/j.cell.2015.05.045 26091042 PMC4615699

[ejn15516-bib-0030] Ogasawara, D. , Ichu, T. A. , Jing, H. , Hulce, J. J. , Reed, A. , Ulanovskaya, O. A. , & Cravatt, B. F. (2019). Discovery and optimization of selective and in vivo active inhibitors of the Lysophosphatidylserine lipase alpha/beta‐hydrolase domain‐containing 12 (ABHD12). Journal of Medicinal Chemistry, 62(3), 1643–1656. 10.1021/acs.jmedchem.8b01958 30720278 PMC6583925

[ejn15516-bib-0031] Pathak, D. , Mehendale, N. , Singh, S. , Mallik, R. , & Kamat, S. S. (2018). Lipidomics suggests a new role for ceramide synthase in phagocytosis. ACS Chemical Biology, 13(8), 2280–2287. 10.1021/acschembio.8b00438 29963848 PMC6102644

[ejn15516-bib-0032] Pilat, A. , Herrnreiter, A. M. , Skumatz, C. M. , Sarna, T. , & Burke, J. M. (2013). Oxidative stress increases HO‐1 expression in ARPE‐19 cells, but melanosomes suppress the increase when light is the stressor. Investigative Ophthalmology & Visual Science, 54(1), 47–56. 10.1167/iovs.12-11153 23221079 PMC3544422

[ejn15516-bib-0033] Rai, A. , Pathak, D. , Thakur, S. , Singh, S. , Dubey, A. K. , & Mallik, R. (2016). Dynein clusters into lipid microdomains on phagosomes to drive rapid transport toward lysosomes. Cell, 164(4), 722–734. 10.1016/j.cell.2015.12.054 26853472 PMC4752818

[ejn15516-bib-0034] Rajendran, A. , Vaidya, K. , Mendoza, J. , Bridwell‐Rabb, J. , & Kamat, S. S. (2020). Functional annotation of ABHD14B, an orphan serine hydrolase enzyme. Biochemistry, 59(2), 183–196. 10.1021/acs.biochem.9b00703 31478652 PMC6976303

[ejn15516-bib-0035] Rueden, C. T. , Schindelin, J. , Hiner, M. C. , DeZonia, B. E. , Walter, A. E. , Arena, E. T. , & Eliceiri, K. W. (2017). ImageJ2: ImageJ for the next generation of scientific image data. BMC Bioinformatics, 18(1), 529. 10.1186/s12859-017-1934-z 29187165 PMC5708080

[ejn15516-bib-0036] Schindelin, J. , Rueden, C. T. , Hiner, M. C. , & Eliceiri, K. W. (2015). The ImageJ ecosystem: An open platform for biomedical image analysis. Molecular Reproduction and Development, 82(7–8), 518–529. 10.1002/mrd.22489 26153368 PMC5428984

[ejn15516-bib-0037] Shanbhag, K. , Mhetre, A. , Khandelwal, N. , & Kamat, S. S. (2020). The Lysophosphatidylserines‐an emerging class of Signalling Lysophospholipids. The Journal of Membrane Biology, 253(5), 381–397. 10.1007/s00232-020-00133-2 32767057 PMC7116134

[ejn15516-bib-0038] Singh, S. , Joshi, A. , & Kamat, S. S. (2020). Mapping the neuroanatomy of ABHD16A, ABHD12, and Lysophosphatidylserines provides new insights into the pathophysiology of the human neurological disorder PHARC. Biochemistry, 59(24), 2299–2311. 10.1021/acs.biochem.0c00349 32462874 PMC7311203

[ejn15516-bib-0039] Steinberg, B. E. , & Grinstein, S. (2008). Pathogen destruction versus intracellular survival: The role of lipids as phagosomal fate determinants. The Journal of Clinical Investigation, 118(6), 2002–2011. 10.1172/JCI35433 18523652 PMC2396921

[ejn15516-bib-0040] Tauber, A. I. (2003). Metchnikoff and the phagocytosis theory. Nature Reviews. Molecular Cell Biology, 4(11), 897–901. 10.1038/nrm1244 14625539

[ejn15516-bib-0041] Tyurina, Y. Y. , Shvedova, A. A. , Kawai, K. , Tyurin, V. A. , Kommineni, C. , Quinn, P. J. , Schor, N. F. , Fabisiak, J. P. , & Kagan, V. E. (2000). Phospholipid signaling in apoptosis: Peroxidation and externalization of phosphatidylserine. Toxicology, 148(2‐3), 93–101. http://www.ncbi.nlm.nih.gov/pubmed/10962127 10962127 10.1016/s0300-483x(00)00199-2

[ejn15516-bib-0042] Viader, A. , Ogasawara, D. , Joslyn, C. M. , Sanchez‐Alavez, M. , Mori, S. , Nguyen, W. , Conti, B. , & Cravatt, B. F. (2016). A chemical proteomic atlas of brain serine hydrolases identifies cell type‐specific pathways regulating neuroinflammation. eLife, 5, e12345. 10.7554/eLife.12345 26779719 PMC4737654

[ejn15516-bib-0043] Vieira, O. V. , Botelho, R. J. , & Grinstein, S. (2002). Phagosome maturation: Aging gracefully. The Biochemical Journal, 366(Pt 3), 689–704. 10.1042/BJ20020691 12061891 PMC1222826

